# Cognitive function in amyotrophic lateral sclerosis: a cross-sectional and prospective pragmatic clinical study with review of the literature

**DOI:** 10.1007/s10072-023-07262-1

**Published:** 2023-12-18

**Authors:** Adamantios Katerelos, Panagiotis Alexopoulos, Polychronis Economou, Panagiotis Polychronopoulos, Elisabeth Chroni

**Affiliations:** 1 Department of Medicine, School of Health Sciences, University of Patras, Patras, Greece; 2 Department of Neurology, Patras University General Hospital, Rio, Greece; 3 Mental Health Services, Patras University General Hospital, Rio, Greece; 4 Medical School, Trinity College Dublin, Global Brain Health Institute, The University of Dublin, Dublin, Republic of Ireland; 5 Faculty of Medicine, Klinikum Rechts Der Isar, Department of Psychiatry and Psychotherapy, Technical University of Munich, Munich, Germany; 6 Patras Dementia Day Care Centre, Patras, Greece; 7 Department of Civil Engineering (Statistics), School of Engineering, University of Patras, Patras, Greece

**Keywords:** Amyotrophic lateral sclerosis, ALS, Cognitive impairment, FAB, MoCA

## Abstract

**Background:**

Amyotrophic lateral sclerosis (ALS) can present with either bulbar or spinal symptoms, and in some cases, both types of symptoms may be present. In addition, cognitive impairment has been observed in ALS. The study aimed to evaluate the frontal and general cognitive performance in ALS not only cross-sectionally but also longitudinally.

**Methods and materials:**

The Frontal Assessment Battery (FAB) and the Montreal Cognitive Assessment (MoCA) were employed to assess cognitive function in 52 adults with ALS and 52 cognitively healthy individuals. The statistical analyses encompassed the Pearson Chi square test, the Skillings-Mack test, the Spearman’s rank correlation coefficient, and the Proportional Odds Logistic Regression Model (POLR).

**Results:**

Cross-sectionally, lower cognitive performance was associated with ALS diagnosis, older age, and motor functional decline. The cognitive impairment of individuals with bulbar and spinal-bulbar symptoms showed faster deterioration compared to those with spinal symptoms. The spinal subgroup consistently performed worst in delayed recall and attention, while the spinal-bulbar and bulbar subgroups exhibited inferior scores in delayed recall, attention, visuospatial skills, orientation, and verbal fluency.

**Conclusion:**

The incorporation of cognitive screening in the diagnostic workup of ALS may be beneficial, as early detection can enhance symptom management and improve the quality of life for both individuals with ALS and their care partners.

**Supplementary Information:**

The online version contains supplementary material available at 10.1007/s10072-023-07262-1.

## Introduction

Amyotrophic lateral sclerosis (ALS) is a devastating progressive neurodegenerative disease. It is characterized by the progressive loss of upper and lower motor neurons in the cortical, bulbar, and spinal regions leading to motor deficits, bulbar palsy, and respiratory insufficiency [1]. The onset of muscle weakness in ALS is usually focal and typically spreads to adjacent body regions [2]. Based on the muscle groups affected at disease onset, ALS can be classified into spinal- or bulbar type [3]. Spinal ALS is characterized by unilateral distal muscle weakness and atrophy in upper or lower limb muscles, while weakness in bulbar muscles with dysarthria or dysphagia, less frequently with dysphonia, or reduced mouth closure or chewing problems is typical for bulbar ALS [4]. Of note, several patients manifest both spinal and bulbar symptoms when the disease diagnosis is established, since there is commonly a 6–18-month diagnostic delay from symptom onset [5]. ALS is an incurable, fatal disease with an average survival time of 2 to 5 years, usually due to respiratory failure [6].

The phenotype of ALS extends beyond motor disturbances. Mounting evidence points out that more than 50% [7] of people with ALS develop executive dysfunction or behavioral deficits, while 15 to 20% of them meet criteria for frontotemporal dementia (FTD) [8, 9]. Cognitive impairment in ALS is not restricted to frontal lobe functions. Impairment in other cognitive domains, including memory and confrontation naming has been reported [10, 11]. Individuals with cognitive deficits early in the disease course tend to be at higher risk for further cognitive decline, whereas people who are cognitively normal at the onset of the disease develop cognitive deficits lately in the disease course [12]. It is noteworthy, that cognitive decline does not seem to follow the pattern of the increasing physical disability and deterioration of spinal and bulbar functions [13].

It is of significance to note that cognitive impairment in ALS is related to brain changes. In people with ALS and cognitive impairment frontal lobe atrophy and lesions along the limbic-thalamo-cortical pathways [14, 15], cortical hypometabolism, particularly in frontotemporal regions and the anterior cingulate gyrus of the adductor gyrus have been reported [16–19]. In addition, cognitive impairment in ALS pertains to frontotemporal brain spongiform changes and endoneuronal ubiquitin inclusions with increased concentration of abnormal TDP-43 aggregates [20–22]. Disruption of neuronal networks, including the default mode network, may also contribute to cognitive impairments in ALS [23].

Most studies focusing on the cognitive function in people with ALS included small samples; the cognitive performance of people with ALS was not compared to people without cognitive impairment; the longitudinal assessment of changes in cognitive function was either absent [24–26] or restricted to periods of less than 12 months [27–29], with the exception of two studies [12, 13]. Nonetheless, detecting cognitive impairment early in its course and monitoring ongoing changes of cognitive symptoms at regular intervals can result in timely initiation of personalized comprehensive care [30], proactive symptoms management, and timely discussions regarding end-of-life preferences. Of note, due to the lack of curative treatment, the main focus of clinicians is on an individualized symptomatic treatment which presupposes a comprehensive diagnostic workup [14].

Our study aimed to shed light on (i) differences in cognitive performance between ALS patient with bulbar, spinal, or both spinal and bulbar involvement compared to people without cognitive impairment, (ii) differences in longitudinal changes in cognitive function in these three clinical subgroups of ALS patients within a follow-up period of 12 months, and on (iii) possible relationships between longitudinal changes of cognitive function and demographics and disease progression.

## Methods and materials

### Study design and ethics approval

The cross-sectional and prospective study was conducted in accordance with the latest revision of the Declaration of Helsinki and was approved by the Scientific Committee on Research and Ethics of the Patras University Hospital (reference number 437/06.09.2018). All participants gave their written informed consent prior their enrolment to the study and after a thorough description of the aims and the procedures of the study.

### Participants

The study sample consisted of individuals with ALS who were assessed/treated at the outpatient unit for neuromuscular diseases of the Department of Neurology of the Patras University Hospital and a convenience sample of individuals without cognitive impairment who served as control and were assessed at the psychogeriatric outpatient unit of the Department of Psychiatry of the above hospital.

Inclusion criteria were medical history, neurologic examination, and electromyographic findings consistent with ALS; absence of neurocognitive disorder; age 18 or older; and treatment/assessment at the Patras University Hospital. Exclusion criteria included (i) neurocognitive disorder (e.g., major neurocognitive disorder due to Alzheimer’s disease, FTD, mild neurocognitive disorder), (ii) serious acute or chronic mental illness (e.g., depression, as indicated by scores higher than seven on the Hamilton Depression Scale, schizophrenia), (iii) treatment with psychoactive drugs or other medication that could affect mental status, (iv) severe motor deficits (upper extremities, dysarthria) that could interfere with neuropsychological performance, and (v) unwillingness to participate in the study. All individuals fulfilled the Revised El Escorial criteria for ALS [16] and were classified into three subgroups as follows based on their symptoms at the time point of their initial assessment at the Patras University Hospital: patients with symptoms and signs restricted to the spinal cord (spinal ALS), patients with isolated bulbar involvement (bulbar ALS), and patients with evidence of both spinal and bulbar involvement (spinal-bulbar ALS). In individuals without cognitive impairment, neither cognitive deficits nor functional impairment was detected based on a thorough diagnostic workup previously described in detail [31].

### Neurocognitive and clinical assessment

The cognitive function of people with ALS was assessed at three different time points. It took place at baseline (the first study visit with each individual, when the diagnosis has already been made), at 6- and 12-month follow-up of individuals with ALS. Demographic data (sex, age, education) were collected during the baseline visit. The assessment relied on the Frontal Assessment Battery (FAB) and the Montreal Cognitive Assessment (MoCA).

Executive functions were assessed with the FAB [32]. The FAB was developed as a screening tool for neurodegenerative diseases with frontal involvement [33–35], and it has been already used in research related to ALS [36–38]. It consists of six subtests, examining frontal functions, such as conceptualization, mental flexibility, motor programming and executive control of action, interference sensitivity, inhibitory control, and environmental autonomy [39]. Abstract reasoning on similarities, lexical fluency, Luria’s “fist-edge-palm” motor series, contradicting instruction, Go-No-Go tasks, and prehension behavior are among the subtests’ contents [40, 41]. Each subtest is graded on a range of 0 to 3, with higher scores pointing to better performance. The sum of these scores yields the total FAB score, which a range from 0 to 18. The complete test takes roughly 10 min to administer [40, 41]. Scores lower than 16 points indicate the presence of mild frontal dysfunction [42].

MoCA was developed as a brief screening test for mild form of cognitive dysfunction like mild neurocognitive disorder, being in many cases a pre-dementia stage of neurodegenerative diseases, and it has been used in research related to ALS [43]. Its administration lasts approximately 10–15 min. The test consists of 12 individual tasks which assess several cognitive domains including visuospatial/executive functions, naming, memory, attention, language, abstraction, delayed recall, and orientation. The maximum total score is 30, while scores lower than 26 and 18 indicate mild and major neurocognitive disorder, respectively [44].

At each visit, disease severity was quantified using the ALS Functional Rating Scale Revised (ALS-FRS-R), which measures 12 aspects of physical function, including one’s ability to swallow and use utensils to climbing stairs and breathing. Each function is scored from 4 (normal) to 0 (no ability), with a maximum total score of 48 and a minimum total score of 0 [45].

### Statistical analysis

In this study, descriptive statistics were computed for all variables, utilizing means, standard deviations, median and range for continuous variables, and counts and percentages for categorical data. The primary statistical methods employed were the Pearson Chi square test, the Skillings-Mack test, and Spearman’s rank correlation coefficient. Additionally, the proportional odds logistic regression model (POLR) was utilized to examine potential predictors of cognitive decline. At the outset, an evaluation of cognitive function was conducted on clinical subgroups comprising individuals with ALS and those without cognitive impairment. Subsequently, a longitudinal analysis of test results was carried out, involving comparisons across three distinct chronological assessments (T0, T1, T2). This analysis was performed on the entire patient sample, as well as on subgroups categorized as spinal, spinal-bulbar, and bulbar. The significance level was set at 0.05. The analysis was carried out in the R environment for statistical computing and visualization.

## Results

### Cognitive function in clinical subgroups of participants with ALS and individuals without cognitive impairment

**Fig. 1:**
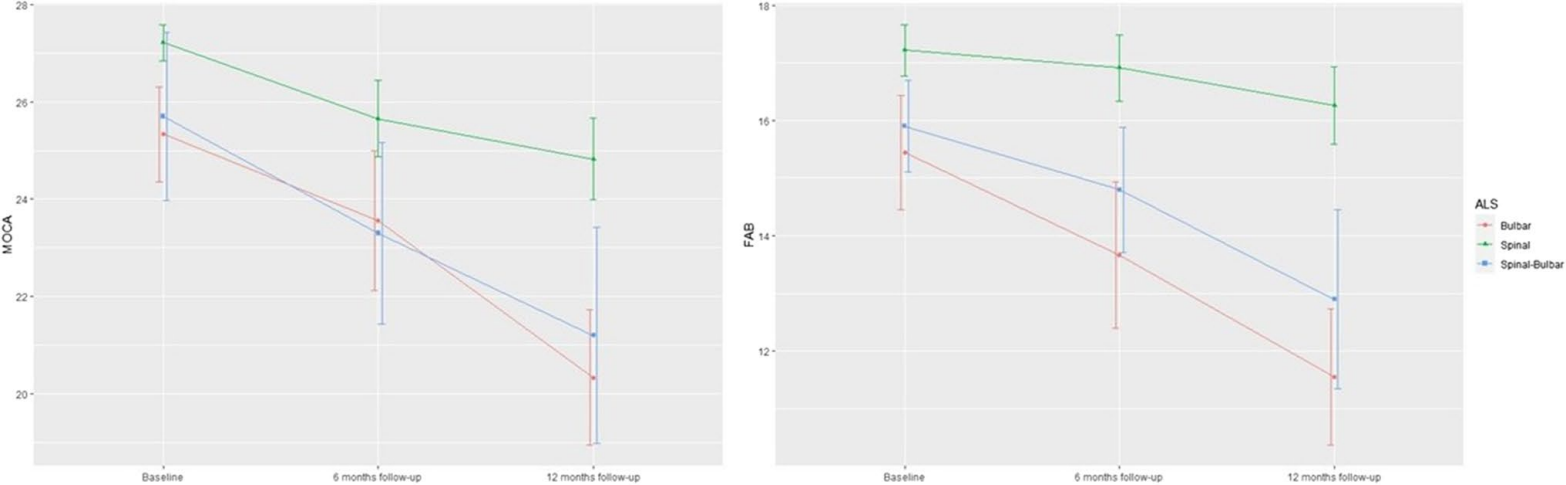
Longitudinal changes of mean performance (with approximate 95% confidence intervals) of individuals with different symptoms of ALS (bulbar, spinal, spinal-bulbar) on MoCA [left (*Y*-axis: total MoCA scores, *X*-axis: the three diferent follow-up time points)] and on FAB [right (*Y*-axis: total fab scores, *X*-axis: the three diferent follow-up time points)]

All participants with ALS and all individuals without cognitive impairment were assessed with MoCA at baseline (Table 1). Performance was abnormal in 11 people with ALS (21%) and only in one individual without ALS (2%). Α statistically significant difference was observed in the MoCA scores between individuals with ALS and those without cognitive impairment (Pearson’s Chi-squared test; *Χ*^2^ = 9.4203, *p* value = 0.002). Regarding the differences between the three subgroups of ALS, in 24 (96%) individuals with spinal symptoms, normal cognitive performance was detected, while normal cognition based on MoCA was observed in seven (58%) participants with bulbar symptoms and in ten (67%) participants with both spinal and bulbar symptoms (Pearson’s Chi-squared test, *Χ*^2^ = 8.772, *p* value = 0.012). There was a slight correlation observed between age and the total score on the MoCA, whereas no correlation was found between education years and the MoCA total score (Spearman rho =  − 0.185; *p* = 0.059 and rho = 0.377; *p* = 0.087, respectively) (see Fig. 1S). According to the results of the logistic regression model, MoCA total score had a significant inverse correlation with all three ALS subgroups (spinal-bulbar; *p* = 0.001, spinal; *p* = 0.006, bulbar; *p* = 0.003), older age (*p* = 0.003), and ALSFRS-R total score (*p* = 0.019) (Table 2).

**Table 1:** Demographic and clinical characteristics of ALS patients and controls

	Spinal subgroup	Bulbar subgroup	Spinal-bulbar subgroup	NCI subgroup
Variables	Mean (sd) – [min–max]	Mean (sd) – [min–max]	Mean (sd) – [min–max]	Mean (sd) – [min–max]
***N*** (baseline)	25	12	15	52
Age (years)	58.6 (9.45) – [42–78]	62.1 (8.34) – [52–80]	63.1 (10.2) – [45–77]	64.9 (8.35) – [42–76]
Sex, female [***N*** (%)]	10 (40%)	5 (42%)	6 (40%)	40 (77%)
Education (years)	11.2 (4.49) – [3–21]	11.8 (2.95) – [6–16]	9.2 (4.02) – [6–18]	13 (3.62) – [4–18]
Time since ALS diagnosis (months)				
Baseline	6 (4.41) – [1–18]	4.5 (3.03) – [1–12]	8.6 (6.79) – [1–4]	N/A
6-month follow-up	11.8 (4.53) – [7–24]	10.4 (3.14) – [7–18]	13.5 (5.49) – [7–24]	N/A
12-month follow-up	17.9 (4.44) – [13–30]	15.8 (1.99) – [13–18]	19.6 (5.10) – [13–27]	N/A
ALSFRS-R ( /48)				
Baseline	42.4 (2.05) – [38–46]	39.3 (2.93) – [34–45]	39.4 (3.94) – [33–44]	N/A
6-month follow-up	40.4 (3.60) – [32–46]	35.5 (3.96) – [32–43]	35.8 (4.69) – [28–44]	N/A
12-month follow-up	38.5 (4.14) – [32–45]	30.2 (2.54) – [26–34]	34.3 (4.35) – [28–41]	N/A
MoCA total score				
Baseline	27 (1.37) – [22–29]	25.3 (1.44) – [22–27]	25.5 (2.45) – [20–29]	28.9 (1.16) – [26-–30]
6-month follow-up (***N*** = 48)	25.7 (1.92) – [22–29] (*N* = 23)	23.6 (2.01) – [21–28] (*N* = 11)	23 (2.57) – [18–27] (*N* = 14)	N/A
12-month follow-up (***N*** = 42)	24.8 (2.03) – [20–29] (*N* = 23)	20.3 (2.12) – [17–25] (*N* = 9)	21.2 (3.58) – [13–24] (*N* = 10)	N/A
FAB total score				
Baseline	17.1 (1.13) – [15–18]	15.3 (1.30) – [13–18]	15.3 (1.90) – [10–18]	16.1 (1.83) – [14–18]
6-month follow-up (***N*** = 48)	16.9 (1.41) – [14–18] (*N* = 23)	13.6 (1.75) – [11–17] (*N* = 11)	14.2 (1.89) – [11–17] (*N* = 14)	N/A
12-month follow-up (***N*** = 42)	16.3 (1.63) – [12–18] (*N* = 23)	11.6 (1.81) – [9–14] (*N* = 9)	12.9 (2.51) – [8–17] (*N* = 10)	N/A

*ALS,* amyotrophic lateral sclerosis; *ALSFRS-R*, Amyotrophic Lateral Sclerosis Functional Rating Scale Revised; *FAB*, Frontal Assessment Battery; *MoCA*, Montreal Cognitive Assessment; *N/A*, data not available; *NCI*, no cognitive impairment

**Table 2:** Results of the logistic ordinal regression model at baseline (T0) which reveal the emergence of factors/predictors that exhibit a detrimental impact on the cognitive profile

	Predictor	coef	S.E	Wald	*p*
MoCA					
	Age	− 0.06588	0.02247	− 2.932	0.003
	Gender	− 0.0229	0.4030	− 0.06	0.955
	Education	0.08233	0.05009	1.644	0.100
	Duration	− 0.685	0.0535	− 1.28	0.200
	Site of onset				
	Spinal-bulbar	− 12.84813	3.99028	− 3.220	0,001
	Spinal	− 11.39533	4.18560	− 2.723	0,006
	Bulbar	− 12.12764	4.02256	− 3.015	0.003
	ALS-FRS-R	0.22871	0.09717	2.354	0.019
FAB					
	Age	− 0.1020	0.02630	− 3.876	0,000
	Gender	− 0.6172	0.4934	− 1.25	0.211
	Education	0.0447	0.0632	0.71	0.479
	Duration	− 0.0953	0.0572	− 1.67	0.096
	Site of onset				
	Spinal-bulbar	− 9.6943	3.53800	− 2.740	0.006
	Spinal	− 7.8788	3.69049	− 2.135	0.033
	Bulbar	− 9.1241	3.50450	− 2.604	0.009
	ALS-FRS-R	0.2083	0.08729	2.387	0.017

*ALSFRS-R*, Amyotrophic Lateral Sclerosis Functional Rating Scale Revised; *FAB*, Frontal Assessment Battery; *MoCA*, Montreal Cognitive Assessment; *Coef* represents the value (in log-odds units) that can be used for the regression equation predicting the dependent from the independent variables; *SE*, standard error; *Wald*. used to test the significance of individual coefficients in the model

FAB data were available from all participants with ALS but only from 24 healthy individuals, because FAB was incorporated into the routine diagnostic workup at the psychogeriatric outpatient unit later than MoCA (Table 1). Abnormal frontal function was observed in 18 (35%) participants with ALS and in ten (42%) individuals without cognitive impairment. The frequency of abnormal frontal function did not vary across the groups (Pearson’s Chi-squared test, *Χ*^2^ = 0.351, *p* value = 0.554). Of note, individuals with spinal ALS had significantly more frequently normal frontal function (88%) compared to the bulbar ALS group and participants with both bulbar and spinal symptoms (33% and 53% respectively) (Pearson’s Chi-squared test, *X*^2^ = 12.0586, *p* value = 0.002). There was a significant correlation observed between age and the total score of the FAB. However, no significant correlation was found between education level and FAB total score (Spearman rho =  − 0.446; *p* = 0.05 and rho = 0.164; *p* = 0.1564, respectively) (see Fig. 2S). According to the results of the logistic regression model, the total FAB scores were found to be inversely correlated with the three ALS subgroups (spinal-bulbar; *p* = 0.006, spinal; *p* = 0.033, bulbar; *p* = 0.009), older age (*p* = 0.000), and ALSFRS-R total score (*p* = 0.017) (Table 2).

### Longitudinal cognitive changes

Significant changes were detected in MoCA scores over time. Forty-two individuals with ALS completed both follow-up assessments (Table 1). Four participants from the second assessment and an additional six participants from the third assessment were lost to follow-up either because of severe physical disabilities, i.e., motor and speech impairment, that impeded their participation in follow-up visits or due to the desire of the patient to discontinue participation. In the entire ALS group, a gradual decline in MoCA total scores over time was observed (Fig. 1 and Table 1S). Significant statistical differences were observed between the first and second assessment (*X*^2^ = 18.4565, *p* = 0.000) and first and third assessment (*X*^2^ = 32.4851, *p* < 0.001) but not between the second and third assessment (*X*^2^ = 2.674, *p* = 0.102). MoCA subscale scores exhibited significant variation across the three assessments, as determined by the mean difference from the score of each subscale. At the first assessment, impairment was significantly poor for the domains of attention and memory, whereas at the next two assessments, scores also showed a significant decrease in delayed recall and verbal fluency (Table 1S). Further analysis of subgroups revealed that the spinal subgroup consistently exhibited the poorest performance in delayed recall and attention domains. Similarly, the spinal-bulbar and bulbar subgroups demonstrated inferior scores in delayed recall and attention domains during the initial assessment. However, in the subsequent two assessments, visuospatial skills and orientation were predominantly associated with lower scores, followed by verbal fluency (Table 1S). Of note, the results of the Skilling Mack test point to significant changes in MoCA total scores analyzing purely the 42 participants with ALS over time (*X*^2^ = 89.738, *p* < 0.001). In addition, the further analysis of MoCA performance significantly decreased over time in people with bulbar symptoms (*X*^2^ = 17.8, *p* = 0.023), with spinal symptoms (*X*^2^ = 35.193, *p* = 0.027), as well as in individuals with spinal-bulbar symptoms (*X*^2^ = 22.709, *p* = 0.007). Moreover, the analysis based on the Skilling-Mack test unveiled significant variation between MoCA performance in all three ALS subgroups and total MoCA scores (bulbar; *X*^2^ = 15.5, *p* < 0.001/spinal; *X*^2^ = 16.786, *p* < 0.001/spinal-bulbar; *X*^2^ = 19.05, *p* < 0.001).

Significant longitudinal changes in FAB scores were also observed. Forty-two individuals with ALS successfully underwent the required follow-up assessments, similar to the MoCA. Total scores significantly declined over time (Table 1), subsequently resulting in an increase in the frequency of ALS individuals with abnormal FAB scores (< 16) (Fig. 1). The findings of the Skilling Mack test illustrate the significant longitudinal change of FAB total scores (*X*^2^ = 102.55,* p* < 0.001). Moreover, a more in-depth analysis of the three distinct chronological assessments revealed a noteworthy statistical disparity between the initial and final assessments (*X*^2^ = 7.1153, *p* = 0.008). Through an analysis of the means and percentages of patients who scored less than three, a decrease in performance on FAB subscale scores was detected except for conceptualization and prehension behavior (Table 2S). The decrease reached its maximum in the following FAB subscales: conflicting instructions, inhibitory control, and verbal fluency (Table 2S). Notably, the subgroup analysis indicated that the spinal subgroup exhibited the greatest decrease in inhibitory control, while the spinal-bulbar subgroup demonstrated the greatest decrease in conflicting instructions. Furthermore, the bulbar subgroup exhibited the greatest decrease in verbal fluency (Table 2S). In addition, the findings of the Skilling Mack test, analyzing purely the 42 participants illustrate that total FAB scores significantly decreased over time in all three ALS subgroups (bulbar; *X*^2^ = 19.6, *p* = 0.012/spinal; *X*^2^ = 43.034, *p* = 0.003/spinal-bulbar; *X*^2^ = 24.727, *p* = 0.003). Nonetheless, statistical significance was observed only in the bulbar- and spinal-bulbar subgroup when comparing FAB performance in all three ALS subgroups and total FAB scores (bulbar; *X*^2^ = 16.056, *p* < 0.001/spinal-bulbar; *X*^2^ = 17.15, *p* < 0.001), indicating slighter changes in individuals with spinal ALS symptoms (*X*^2^ = 3.930, *p* = 0.140) compared to the two subgroups with bulbar symptoms.

## Discussion

The exact mechanisms underlying cognitive impairment in ALS are not yet fully understood. However, several hypotheses have been proposed. One hypothesis suggests that the degenerative process in ALS affects not only the motor neurons but also other regions of the central nervous system, including the frontal and temporal lobes, which are crucial for cognitive function [46]. Another hypothesis proposes that the accumulation of abnormal proteins, such as TDP-43, in the brain contributes to cognitive dysfunction in ALS [47]. Additionally, neuroinflammation, excitotoxicity, and oxidative stress have also been implicated in the cognitive impairment observed in ALS [12].

The present study sheds light on cognitive function in people with different symptoms of ALS. The presence of bulbar ALS symptoms was related to more severe cognitive deficits compared to individuals with only spinal symptoms, while all three subgroups of people with ALS and different symptom categories experienced a significant decline in their cognitive function over time. The novelty of the study includes the classification of people with ALS into groups based on their clinical symptoms at the time point of ALS, the cross-sectional comparison of their cognitive performance to that of individuals without cognitive impairment, as well as the study of longitudinal changes of cognitive function in individuals with ALS within a follow-up period of 12 months.

According to the literature, patients with bulbar onset ALS showed more significant deficits in executive function than patients with spinal onset [13, 48, 49], which was also demonstrated in our study. It has been found that the occurrence of bulbar symptoms is associated with more pronounced deterioration of motor functions [50] and cognitive impairment [28, 51], findings that are fully consistent with the findings of our own research. With rare exceptions [52], it is very likely that the development of bulbar symptoms will alter cognitive function during the course of the disease [28, 53]. A hypothesis posits that the proximity of the cortical regions that regulate facial and bulbar muscles to the prefrontal cognitive cortex may facilitate the spread of TDP-43 lesions [54]. Clinical observations of patients diagnosed with amyotrophic lateral sclerosis (ALS) have unveiled a notable correlation between the advancement of this debilitating condition and an increase in p-TDP-43 lesions, accompanied by the degeneration of motor neurons [55]. The dissemination of TDP-43 protein is observed in various neurodegenerative disorders, as a growing body of evidence from clinical research suggests that the spread of misfolded TDP-43 aggregates is intricately linked to the advancement and intensity of neurodegenerative diseases [54, 56]. Nevertheless, the precise molecular mechanism responsible for the propagation of TDP-43 remains elusive.

Performance of individuals with ALS on MoCA- and FAB subtasks sheds light on the cognitive phenotype of ALS. Impairment was noted in almost all FAB subtasks, most notably in conflicting instructions, inhibitory control, and verbal fluency. Previous studies have also found verbal fluency to be among the lowest FAB subscale scores [25, 26] in ALS patients. Performance on prehension behavior and conceptualization were almost universally preserved. As far as MoCA, performance in our sample was comparatively inferior in the domains of memory and attention, as well as verbal fluency. Furthermore, in the last assessment, visuospatial skills and orientation appeared to be adversely impacted, particularly among bulbar and spinal-bulbar groups. It is noteworthy to mention that memory impairment is one of the most consistently reported cognitive deficiencies in patients with ALS, with immediate and delayed verbal recall more frequently affected [10, 11, 34, 48]. Poor performance on “non-executive” MoCA tasks may be an indirect consequence of disordered higher-order frontal lobe functions. Selective impairment of recall relative to recognition abilities suggests that disordered retrieval processes secondary to frontal lobe-dependent attentional dysfunction underlie memory impairment in ALS [10, 48]. This hypothesis is supported by previous reports of impaired attention span, concentration, and working memory in ALS patients [34, 48]. Deficits in these domains may thus account for poor performance on multiple MoCA subscale such as memory and attention something which was showed in our study.

According to the findings of the employed regression models, both older age and ΑLS symptom severity are inversely related to cognitive function. Previous reports point out that cognitive decline in ALS is associated with old age, low education level, severity of the disease measured with the ALS-FRS-r scale, C9orf72 gene mutation, and family history of dementia [46, 57, 58]. In addition, older age has been found more likely to be associated with cognitive decline over time [28, 52]. Interestingly, a recent study [28] showed that there is a longitudinal worsening of cognitive impairment which correlates with disease severity measured by the ALS-FRS-R scale, a finding that is in contrast with other reports suggesting no relationship between longitudinal changes of ALS symptoms and changes in cognitive performance.

Behavioral impairment in ALS is a significant aspect of the disease. Studies have shown that mild-to-moderate behavioral changes have been reported in a wide range of ALS patients, ranging from 17 to 88% [49]. This variability in reported percentages is likely due to differences in the methods used to detect and assess behavioral deficits. However, there is limited knowledge about how changes in behavior develop over the course of ALS. Disinhibition and apathy are the most common progressive behavioral alterations observed, often occurring alongside cognitive impairment. These changes have been linked to a negative prognosis in approximately 50% of ALS individuals [7, 28, 59]. Additionally, research has found that frustration tolerance worsens, adaptability to new situations or changing opinions decreases, and insight diminishes after a 1-year follow-up [59]. It is important to note that behavioral data in ALS studies are typically obtained from caregivers through structured questionnaires and interviews. Therefore, it is possible that caregivers’ perspectives may be influenced by various factors such as psychosocial stress, mood, or their attitude towards adapting to changes over time [29]. To better understand these potential confounding effects, further studies with longer follow-up periods are needed. These studies would help unravel the complexities surrounding behavioral changes in ALS patients.

Genetic factors play a crucial role in the cognitive and behavioral impairment observed in ALS. Mutations in several genes, namely chromosome 9 open reading frame 72 (C9orf72) gene, tank-binding kinase 1 (TBK1), sequestosome-1 (SQSTM1), TAR DNA-binding protein (TARDBP), valosin-containing protein (VCP), coiled-coil-helix-coiled-coil-helix domain-containing protein 10 (CHCHD10), and sequestosome-1 (SQSTM1) can contribute to cognitive impairment and are closely associated with both ALS and FTD [60–62]. It is estimated that 70% of individuals with familiar ALS have an identified genetic mutation [60], while the cause of sporadic ALS, which accounts for the majority of cases, likely involves a combination of genetic and environmental factors [62]. Identifying these genetic determinants is essential for comprehending the prognosis of ALS, the development of targeted interventions, and the improvement of cognitive and behavioral symptom management in ALS patients [60–62].

The present study had several limitations. First, the size of the each of the three ALS subgroups was relatively small. Nevertheless, differences in performance in various cognitive domains between groups reached statistical significance. Second, we did not perform a comprehensive neuropsychological array on our patients by addressing the Edinburgh Cognitive Behavioural ALS Screen (ECAS) which is considered the gold standard for individuals with ALS. Unfortunately, the ECAS had not been published at the beginning of our study and had not been validated in Greek population. Of note, the utility of ECAS may not always be high in detecting deficits in executive function of individuals with ALS [63]. Nevertheless, two recent studies [38, 43] show that both FAB and MoCA have excellent sensitivity and good specificity in detecting cognitive impairment in ALS. Third, we did not assess behavioral changes in patients because neither FAB nor MoCA evaluates behavioral function. Forth, performance on MoCA and FAB in individuals with ALS may be affected by severe motor impairments, such as hand weakness and speech impairment. Thus, our findings may have been biased by motor impairments. In addition, the concentration of neurofilaments (NfL) and phosphorylated NfH (pNFH) in cerebrospinal fluid and/or serum, which have emerged as diagnostic biomarkers of ALS and as markers of the progression of clinical symptoms of ALS (cognitive and motor performance) and may contribute to diagnostic accuracy, were not considered in our study [64]. Moreover, the majority of patients refrained from undergoing genetic testing. This can be attributed to two primary reasons: patient refusal based on concerns regarding potential psychological and moral implications for both themselves and their families, or the limited accessibility and high cost of genetic testing, which is contingent upon healthcare resources. It is important to acknowledge that FAB was completed only by a part of study participants without cognitive impairment. Finally, since the clinical diagnoses of ALS are not always confirmed at autopsy. Hence, possibly erroneous clinical assessments should be also taken into account [65].

## Conclusion

Cognitive impairment is an integral feature of ALS patients. Early identification of cognitive change indicators allows the provision of information to patients and care partners regarding the presence of even mild cognitive deficits. The estimation of the risk for further cognitive decline contributes to the development of personalized symptom management plans for individuals with a disease for which no causal therapy is available yet. Neuroimaging techniques and other biomarkers could help to group in individuals with ALS according to their risk of cognitive impairment [66–68]. For instance, cognitive performance correlates with changes in diffusion tensor imaging (DTI) [69], while modified neuropsychological tests or alternative methods of cognitive testing, such as those with eye-tracker controlled or brain-computer interface [70, 71], could be used to screen patients since the by-pass potential biases stemming from severe motor dysfunction. Furthermore, it is of importance that future studies take into account the effects of medical, neurological, psychiatric, and pharmacological factors that may affect the cognitive performance of individuals with ALS and, if possible, to conduct multi-centre longitudinal studies safeguarding larger study samples.

### Supplementary Information

Below is the link to the electronic supplementary material.

Supplementary file1 (DOCX 502 KB)

## Data Availability

The data presented in this study are available upon request from the corresponding author.
