# Correction to: Cognitive function in amyotrophic lateral sclerosis: a cross‑sectional and prospective pragmatic clinical study with review of the literature

**DOI:** 10.1007/s10072-024-07408-9

**Published:** 2024-02-19

**Authors:** Adamantios Katerelos, Panagiotis Alexopoulos, Polychronis Economou, ·Panagiotis Polychronopoulos, Elisabeth Chroni

**Affiliations:** 1 Department of Medicine, School of Health Sciences, University of Patras, Patras, Greece; 2 Department of Neurology, Patras University General Hospital, Rio, Greece; 3 Mental Health Services, Patras University General Hospital, Rio, Greece; 4 Medical School, Trinity College Dublin, Global Brain Health Institute, The University of Dublin, Dublin, Republic of Ireland; 5 Faculty of Medicine, Klinikum Rechts Der Isar, Department of Psychiatry and Psychotherapy, Technical University of Munich, Munich, Germany; 6 Patras Dementia Day Care Centre, Patras, Greece; 7 Department of Civil Engineering (Statistics), School of Engineering, University of Patras, Patras, Greece

**Correction to: Neurological Sciences (2024)**

10.1007/s10072-023-07262-1

The original article contains an error in Table 1. First, in the first column (variables), change "Education (months)" to "Education (years)". Secondly, at the end of the same table, where some terms are explained, change "*MoC*, Montreal Cognitive Assessment" to "*MoCA*, Montreal Cognitive Assessment".

The original article has been corrected.

