# Supplementary material for: Cognitive function in amyotrophic lateral sclerosis: a cross-sectional and prospective pragmatic clinical study with review of the literature
Source: Neurol Sci. 2023 Dec 18;45(5):2075–85. doi: 10.1007/s10072-023-07262-1 (PMC11021277; doi:10.1007/s10072-023-07262-1)
Supplement: Supplementary file 1 — Supplementary file1 (DOCX 502 KB) [file 10072_2023_7262_MOESM1_ESM.docx]

Article

**Cognitive function in Amyotrophic Lateral Sclerosis: A cross-sectional and prospective pragmatic clinical study with review of the literature.**

**Adamantios Katerelos^1^, Panagiotis Alexopoulos^2,3,4,5^, Polychronis Economou^6^, Panagiotis Polychronopoulos^1^ and Elisabeth Chroni^1^**

^1^Department of Neurology, Patras University General Hospital, Department of Medicine, School of Health Sciences, University of Patras, Patras, Greece

^2^Mental Health Services, Patras University General Hospital, Department of Medicine, School of Health Sciences, University of Patras, Patras, Greece

^3^Global Brain Health Institute, Medical School, Trinity College Dublin, The University of Dublin, Dublin, Republic of Ireland

^4^Department of Psychiatry and Psychotherapy, Klinikum rechts der Isar, Faculty of Medicine, Technical University of Munich, Munich, Germany

^5^Patras Dementia day care centre, Patras, Greece

^6^Department of Civil Engineering (Statistics), School of Engineering, University of Patras, Patras, Greece

**Figures and Tables**

This supplementary material comprises tables and figures that are cited in the main text of the paper, enhancing further the clarity and depth of the presented results.

**
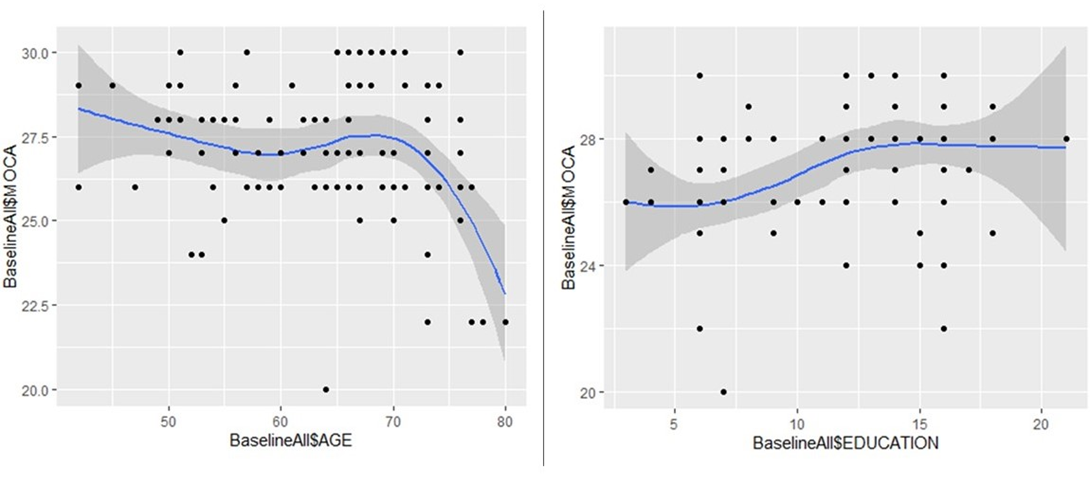
Fig. 1S.** Correlation between MoCA total score with age (left / Y-axis: total MoCA scores, X-axis: age in years) and with education years (right / Y-axis: total MoCA scores, X-axis: education in years) at the first assessment of all participants, healthy and no healthy.

**
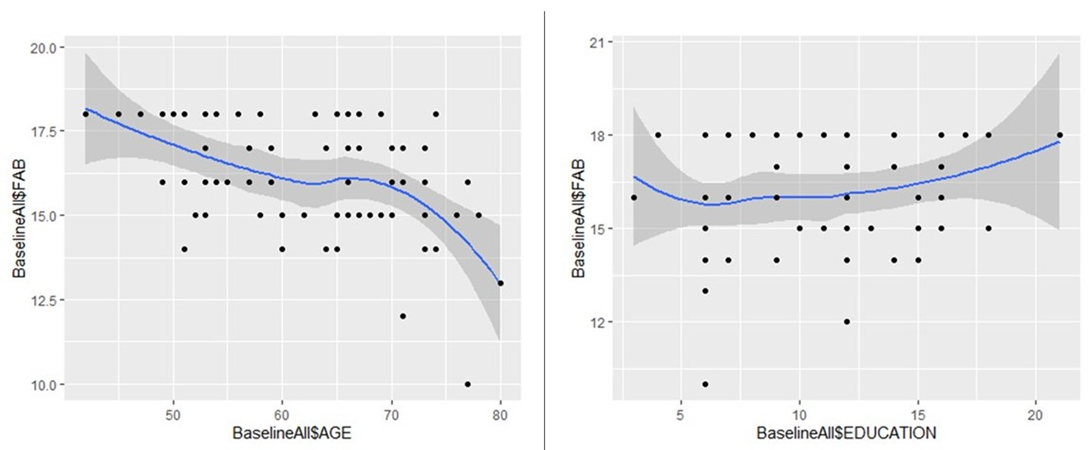
Fig. 2S*.*** Correlation between FAB total score with age (left / Y-axis: total FAB scores, X-axis: age in years) and with education years (right / Y-axis: total FAB scores, X-axis: education in years) at the first assessment of all participants, healthy and no healthy

**Table 1S.** Summary of MoCA subscales’ results per assessment of participants with ALS by subgroup (numbers in brackets next to each subscale indicate the maximum subscale scores).

| ENTIRE SAMPLE | Initial assessment – T0  mean (sd) – difference* – median (range) | Second assessment – T1  mean(sd)–difference*–median (range) | Third assessment – T2  mean(sd)–difference* –median (range) |
| --- | --- | --- | --- |
| N | 52 | 48 | 42 |
| Visuospatial skills [5] | 4.73 (0.45) – [-0.27] – 5 (4-5) | 4.63 (0.61) – [-0.36] – 5 (3-5) | 4.43 (0.94) – [-0.57] – 5 (2-5) |
| Naming [3] | 2.79 (0.41) – [-0.21] – 3 (2-3) | 2.77 (0.42) – [-0.23] – 3 (2-3) | 2.74 (0.46) – [-0.26] – 3 (2-3) |
| Attention (Digit Span) [2] | 1.96 (0.19) – [-0,04] – 2 (1-2) | 1.94 (0.24) – [-0.06] – 2 (1-2) | 1.95 (0.22) – [-0.05] – 2 (1-2) |
| Attention (Vigilance) [1] | 0.40 (0.50) – [-**0.60**] – 0 (0-1) | 0.33 (0.48) – [**-0.67**] – 0 (0-1) | 0.10 (0.30) – [**-0.90**] – 0 (0-1) |
| Attention (Serial 7s) [3] | 2.50 (0.50) – [**-0.50**] – 2.5 (2-3) | 2.40 (0.49) – [**-0.60**] – 2 (2-3) | 2.29 (0.64) – [**-0.71**] – 2 (0-3) |
| Sentence Repetition [2] | 2.00 (0.00) – [-0.00] – 2 (2-2) | 2.00 (0.00) – [ 0.00] – 2 (2-2) | 2.00 (0.00) – [-0.00] – 2 (2-2) |
| Verbal Fluency [1] | 0.77 (0.43) – [-0.23] – 1 (0-1) | 0.35 (0.48) – [**-0.65**] – 0 (0-1) | 0.31 (0.47) – [**-0.69**] – 0 (0-1) |
| Abstraction [2] | 1.98 (0.14) – [-0.02] – 2 (1-2) | 1.98 (0.14) – [-0.02] – 2 (1-2) | 1.98 (0.15) – [-0.02] – 2 (1-2) |
| Delayed Recall [5] | 2.48 (0.99) – [**-2.52**] – 3 (0-4) | 1.50 (1.07) – [**-3.50**] – 1.5 (1-2) | 1.07 (0.99) – [**-3.93**] – 1 (0-3) |
| Orientation [6] | 5.85 (0.41) – [-0.15] – 6 (4-6) | 5.75 (0.48) – [-0.25] – 6 (4-6) | 5.43 (0.67) – [-0.57] – 6 (4-6) |
| SPINAL SUBGROUP | | | |
| N | 25 | 23 | 23 |
| Visuospatial skills [5] | 4.88 (0.33) – [-0.12] – 5 (4-5) | 4.87 (0.34) – [-0.13] – 5 (3-5) | 4.91 (0.29) – [-0.09] – 5 (4-5) |
| Naming [3] | 2.76 (0.44) – [-0.24] – 3 (2-3) | 2.78 (0.42) – [-0.22] – 3 (2-3) | 2.78 (0.42) – [-0.22] – 3 (2-3) |
| Attention (Digit Span) [2] | 2.00 (0.00) – [-0.00] – 2 (2-2) | 2.00 (0.00) – [-0.00] – 2 (2-2) | 2.00 (0.00) – [-0.00] – 2 (2-2) |
| Attention (Vigilance) [1] | 0.44 (0.51) – [**-0.56**] – 0 (0-1) | 0.39 (0.50) – [**-0.61**] – 0 (0-1) | 0.17 (0.39) – [**-0.83**] – 0 (0-1) |
| Attention (Serial 7s) [3] | 2.40 (0.50) – [**-0.60**] – 2 (2-3) | 2.43 (0.51) – [**-0.57**] – 2 (2-3) | 2.43 (0.59) – [**-0.57**] – 2 (1-3) |
| Sentence Repetition [2] | 2.00 (0.00) – [-0.00] – 2 (2-2) | 2.00 (0.00) – [ 0.00] – 2 (2-2) | 2.00 (0.00) – [-0.00] – 2 (2-2) |
| Verbal Fluency [1] | 0.88 (0.33) – [-0.12] – 1 (0-1) | 0.52 (0.51) – [-0.48] – 1 (0-1) | 0.43 (0.51) – [-**0.57**] – 0 (0-1) |
| Abstraction [2] | 2.00 (0.00) – [-0.00] – 2 (2-2) | 2.00 (0.00) – [-0.00] – 2 (2-2) | 2.00 (0.00) – [-0.00] – 2 (2-2) |
| Delayed Recall [5] | 2.92 (0.78) – [**-2.08**] – 3 (1-4) | 2.00 (1.04) – [**-3.00**] – 2 (0-4) | 1.52 (0.99) – [**-3.48**] – 2 (0-3) |
| Orientation [6] | 5.96 (0.20) – [-0.04] – 6 (5-6) | 5.91 (0.29) – [-0.09] – 6 (5-6) | 5.83 (0.39) – [-0.17] – 6 (5-6) |
| SPINAL-BULBAR SUBGROUP | | | |
| N | 15 | 14 | 10 |
| Visuospatial skills [5] | 4.53 (0.52) – [-0.47] – 5 (4-5) | 4.28 (0.73) – [**-0.72**] – 4 (3-5) | 4.10 (1.10) – [**- 0.90**] – 4.5 (2-5) |
| Naming [3] | 2.80 (0.41) – [-0.20] – 3 (2-3) | 2.79 (0.43) – [-0.21] – 3 (2-3) | 2.70 (0.48) – [- 0.30] – 3 (2-3) |
| Attention (Digit Span) [2] | 1.93 (0.26) – [-0.07] – 2 (1-2) | 1.79 (0.43) – [-0.21] – 2 (1-2) | 1.80 (0.42) – [- 0.20] – 2 (1-2) |
| Attention (Vigilance) [1] | 0.27 (0.46) – [**-0.73**] – 0 (0-1) | 0.14 (0.36) – [**-0.86**] – 0 (0-1) | 0.00 (0.00) – [**- 1.00**] – 0 (0-0) |
| Attention (Serial 7s) [3] | 2.73 (0.50) – [-0.27] – 3 (2-3) | 2.36 (0.50) – [**-0.64**] – 2 (2-3) | 2.20 (0.92) – [**- 0.80**] – 2 (0-3) |
| Sentence Repetition [2] | 2.00 (0.00) – [-0.00] – 2 (2-2) | 2.00 (0.00) – [ 0.00] – 2 ( 2-2) | 2.00 (0.00) – [- 0.00] – 2 (2-2) |
| Verbal Fluency [1] | 0.60 (0.51) – [-0.40] – 1 (0-1) | 0.21 (0.43) – [**-0.79**] – 0 (0-1) | 0.20 (0.42) – [**- 0.80**] – 0 (0-1) |
| Abstraction [2] | 2.00 (0.00) – [-0.00] – 2 (2-2) | 2.00 (0.00) – [-0.00] – 2 (2-2) | 2.00 (0.15) – [- 0.00] – 2 (2-2) |
| Delayed Recall [5] | 2.40 (1.21) – [**-2.60**] – 3 (0-4) | 1.00 (0.96) – [**-4.00**] – 1 (0-3) | 0.70 (0.82) – [ **4.30** ] – 0.5 (0-2) |
| Orientation [6] | 5.60 (0.63) – [-0.40] – 6 (4-6) | 5.57 (0.65) – [-0.43] – 6 (4-6) | 4.90 (0.74) – [**- 1.10**] – 5 (4-6) |
| BULBAR SUBGROUP | | | |
| N | 12 | 11 | 9 |
| Visuospatial skills [5] | 4.67 (0.49) – [-0.33] – 5 (4-5) | 4.55 (0.69) – [-0.45] – 5 (3-5) | 3.56 (1.13) – [**-1.44**] – 4 (2-5) |
| Naming [3] | 2.83 (0.39) – [-0.17] – 3 (2-3) | 2.73 (0.47) – [-0.27] – 3 (2-3) | 2.67 (0.50) – [-0.33] – 3 (2-3) |
| Attention (Digit Span) [2] | 1.92 (0.29) – [-0.08] – 2 (1-2) | 2.00 (0.00) – [-0.00] – 2 (2-2) | 2.00 (0.00) – [-0.00] – 2 (2-2) |
| Attention (Vigilance) [1] | 0.50 (0.52) – [**-0.50**] – 0.5 (0-1) | 0.45 (0.52) – [**-0.55**] – 0 (0-1) | 0.00 (0.00) – [**-1.00**] – 0 (0-0) |
| Attention (Serial 7s) [3] | 2.42 (0.51) – [**-0.58**] – 2 (2-3) | 2.36 (0.50) – [**-0,64**] – 2 (2-3) | 2.00 (0.00) – [**-1.00**] – 2 (2-2) |
| Sentence Repetition [2] | 2.00 (0.00) – [-0.00] – 2 (2-2) | 2.00 (0.00) – [ 0.00] – 2 (2-2) | 2.00 (0.00) – [-0.00] – 2 (2-2) |
| Verbal Fluency [1] | 0.75 (0.45) – [-0.25] – 1 (0-1) | 0.18 (0.40) – [**-0.82**] – 0 (0-1) | 0.11 (0.33) – [**-0.89**] – 0 (0-1) |
| Abstraction [2] | 1.92 (0.29) – [-0.08] – 2 (1-2) | 1.91 (0.30) – [-0.09] – 2 (1-2) | 1.89 (0.33) – [-0.11] – 2 (1-2) |
| Delayed Recall [5] | 1.67 (0.78) – [**-3.33**] – 2 (0-3) | 1.09 (0.83) – [**-3.91**] – 1 (0-2) | 0.33 (0.50) – [**-4.67**] – 0 (0-1) |
| Orientation [6] | 5.92 (0.29) – [-0.08] – 6 (5-6) | 5.64 (0.50) – [-0.36] – 6 (5-6) | 5.00 (0.50) – [**-1.00**] – 5 (4-6) |
| *^⁎^ Mean difference from maximum each subscale score.* | | | |

**Table 2S.** Summary of FAB subscales’ results per assessment of participants with ALS by subgroup (maximum score attainable for each subscale is 3).

|  | Initial assessment – T0  mean (sd) – median (range) –  % patients scoring < 3 | Second assessment – T1  mean (sd) – median (range) –  % patients scoring < 3 | Third assessment – T2  mean (sd) – median (range) –  % patients scoring < 3 |
| --- | --- | --- | --- |
| ENTIRE SAMPLE | | | |
| N | 52 | 48 | 42 |
| Conceptualization | 2.98 (0.14) – 3 ( 2-3 ) – 2% | 2.98 (0.14) – 3 (2-3) – 2% | 2.98 (0.15) – 3 ( 2-3 ) – 2% |
| Verbal fluency | 2.54 (0.67) – 3 ( 1-3 ) – **37%** | 2.21 (0.80) – 2 (1-3) – **56%** | 1.98 (0.98) – 2 ( 0-3 ) – **62%** |
| Motor programming | 2.63 (0.60) – 3 ( 1-3 ) – 32% | 2.42 (0.68) – 3 (1-3) – 48% | 2.19 (0.77) – 2 ( 1-3 ) – 60% |
| Conflicting instructions | 2.52 (0.54) – 3 ( 1-3 ) – **46%** | 2.38 (2.38) – 2 (1-3) – **58 %** | 2.19 (0.71) – 2 ( 1-3 ) – **64%** |
| Inhibitory control | 2.56 (0.54) – 3 ( 1-3 ) – **42%** | 2.46 (0.58) – 2.5 (1-3) – **50%** | 2.19 (0.63) – 2 ( 1-3 ) – **69%** |
| Prehension behavior | 2.94 (0.24) – 3 ( 2-3 ) – 6% | 2.90 (0.31) – 3 (2-3) – 10% | 2.93 (0.26) – 3 ( 2-3 ) – 7% |
| SPINAL SUBGROUP |  |  |  |
| N | 25 | 23 | 23 |
| Conceptualization | 3.00 (0.00) – 3 ( 3-3 ) – 0% | 3.00 (0.00) – 3 ( 3-3 ) – 0% | 3.00 (0.00) – 3 ( 3-3 ) – 0% |
| Verbal fluency | 2.88 (0.44) – 3 ( 1-3 ) – 8% | 2.74 (0.54) – 3 ( 1-3 ) – 22% | 2.61 (0.66) – 3 ( 1-3 ) – 30% |
| Motor programming | 2.88 (0.33) – 3 ( 2-3 ) – 12% | 2.74 (0.45) – 3 ( 2-3 ) – 26% | 2.61 (0.50) – 3 ( 2-3 ) – 39% |
| Conflicting instructions | 2.68 (0.48) – 3 ( 2-3 ) – **32%** | 2.74 (0.45) – 3 ( 2-3 ) – **26%** | 2.61 (0.50) – 3 ( 2-3 ) – **39%** |
| Inhibitory control | 2.68 (0.48) – 3 ( 2-3 ) – **32%** | 2.70 (0.47) – 3 ( 2-3 ) – **30%** | 2.49 (0.59) – 3 ( 1-3 ) – **48%** |
| Prehension behavior | 3.00 (0.00) – 3 ( 3-3 ) – 0% | 3.00 (0.00) – 3 ( 3-3 ) – 0% | 2.95 (0.21) – 3 ( 2-3 ) – 4% |
| SPINAL-BULBAR SUBGROUP |  |  |  |
| N | 15 | 14 | 10 |
| Conceptualization | 3.00 (0.00) – 3 ( 3-3 ) – 0% | 3.00 (0.00) – 3 ( 3-3 ) – 0% | 3.00 (0.00) – 3 ( 3-3 ) – 0% |
| Verbal fluency | 2.40 (0.63) – 2 ( 1-3 ) – 53% | 1.86 (0.63) – 2 ( 1-3 ) – **86%** | 1.40 (0.84) – 2 ( 0-2 ) – **100%** |
| Motor programming | 2.47 (0.74) – 3 ( 1-3 ) – 40% | 2.14 (0.77) – 2 ( 1-3 ) – 64% | 1.90 (0.88) – 2 ( 1-3 ) – 70% |
| Conflicting instructions | 2.13 (0.52) – 2 ( 1-3 ) – **80%** | 2.00 (0.39) – 2 ( 1-3 ) – **93%** | 1.80 (0.63) – 2 ( 1-3 ) – **90%** |
| Inhibitory control | 2.40 (0.63) – 2 ( 1-3 ) – **53%** | 2.21 (0.58) – 2 ( 1-3 ) – 71% | 1.90 (0.57) – 2 ( 1-3 ) – 90% |
| Prehension behavior | 2.87 (0.35) – 3 ( 2-3 ) – 13% | 2.86 (0.36) – 3 ( 2-3 ) – 14% | 2.90 (0.32) – 3 ( 2 -3 ) – 10% |
| BULBAR SUBGROUP |  |  |  |
| N | 12 | 11 | 9 |
| Conceptualization | 2.91 (0.29) – 3 ( 2-3 ) – 8% | 2.90 (0.30) – 3 ( 2-3 ) – 9% | 2.89 (0.33) – 3 ( 2-3 ) – 11% |
| Verbal fluency | 2.00 (0.74) – 2 ( 1-3 ) –  **75%** | 1.54 (0.69) – 1 ( 1-3 ) – **91%** | 1.00 (0.50) – 1 ( 0-2 ) – **100%** |
| Motor programming | 2.33 (0.65) – 2 ( 1-3 ) – **58%** | 2.09 (0.70) – 2 ( 1-3 ) – 73% | 1.44 (0.53) – 1 ( 1-2 ) – **100%** |
| Conflicting instructions | 2.67 (0.49) – 3 ( 2-3 ) – 33% | 2.09 (0.54) – 2 ( 1-3 ) – **82%** | 1.56 (0.53) – 2 ( 1-2 ) – **100%** |
| Inhibitory control | 2.50 (0.52) – 2.5 ( 2-3 ) – 50% | 2.27 (0.65) – 2 ( 1-3 ) – 64% | 1.78 (0.44) – 2 ( 1-2 ) – **100%** |
| Prehension behavior | 2.91 (0.29) – 3 ( 2-3 ) – 8% | 2.73 (0.47) – 3 ( 2-3 ) – 27% | 2.89 (0.33) – 3 ( 2-3 ) – 11% |
